# Editorial Comment: Adjustment to an Appropriate Bedtime Improves Nocturia in Older Adults: A Crossover Study

**DOI:** 10.1111/iju.70095

**Published:** 2025-05-06

**Authors:** Tomohiro Matsuo, Ryoichi Imamura

**Affiliations:** ^1^ Department of Urology, Graduate School of Biomedical Sciences Nagasaki University Nagasaki Japan

This study by Okumura et al. [1] offers a novel behavioral strategy to manage nocturia in older adults by adjusting bedtime based on wearable sleep–wake data. It is a timely and well‐designed study that addresses a common, quality‐of‐life‐reducing issue in geriatric care.

The standout feature is the use of a personalized bedtime algorithm, based on actigraphy and the steepest descent method. By correcting sleep timing, the authors achieved measurable improvements in nocturnal urinary frequency (NUF), nocturnal urine volume (NUV), and sleep quality [1]. This intervention avoids medication‐related risks, such as falls or cognitive side effects, which are especially concerning in older populations. The crossover design with a washout period strengthens the validity of results. The significant changes in hours of undisturbed sleep (HUS) and reduced NUV per hour further suggest that personalized sleep timing could meaningfully reduce nocturia symptoms.

The small sample size (*n* = 24) limits generalizability. All participants were Japanese, and cultural or lifestyle factors may influence outcomes. Although the authors discuss hormonal involvement (e.g., melatonin, aldosterone), no biochemical data were collected. Prior studies have shown that behavioral sleep interventions can reduce NUF in older adults with insomnia [2], and that personalized sleep modeling from actigraphy can yield accurate estimations [3]. Melatonin levels are also relevant. Reduced secretion has been linked with nocturia, and exposure to daylight may enhance its production [4]. Sleep restriction has been associated with suppressed renin‐angiotensin‐aldosterone activity, possibly contributing to nocturnal polyuria [5].

This approach can potentially be expanded to broader groups, such as those with overactive bladder or circadian rhythm disorders. Incorporating such strategies into mobile health apps could offer scalable, drug‐free management options. More research is needed to explore the physiological mechanisms involved and long‐term adherence.

Okumura et al. [1] demonstrate that a simple, personalized, non‐pharmacologic intervention can significantly improve nocturia and sleep quality in older adults. Their findings provide a promising step forward in behavioral urology and digital health.

## Author Contributions

**Tomohiro Matsuo:** writing – original draft, review, and editing. **Ryoichi Imamura:** editing and supervision.

## Conflicts of Interest

The authors declare no conflicts of interest.
